# The Impact of COVID-19 on Physical Health Monitoring of Community Rehabilitation Team Patients in NHS Borders

**DOI:** 10.1192/bjo.2022.428

**Published:** 2022-06-20

**Authors:** Rachel Ball, Joanna Bredski

**Affiliations:** NHS Borders, Galashiels, United Kingdom

## Abstract

**Aims:**

People with schizophrenia have a life expectancy that is 10–20 years shorter than the general population. The high incidence of metabolic syndrome, cardiovascular disease and diabetes mellitus in this patient group are thought to be major – and potentially modifiable – factors contributing to this premature mortality. Therefore, annual monitoring of physical health parameters is recommended by organisations including the Scottish Intercollegiate Guidelines Network (SIGN). The SIGN guideline on schizophrenia advises that the following parameters are checked annually for patients with schizophrenia who are on anti-psychotics: ECG, blood glucose, lipid profile, prolactin, BMI/weight, smoking status and blood pressure. Traditionally, this monitoring is overseen in the community by general practitioners. This audit aimed to capture how the COVID-19 pandemic impacted on the annual physical health monitoring of patients under the care of NHS Borders Community Rehabilitation Team.

**Methods:**

A retrospective audit was performed by reviewing notes of the 100 patients on the NHS Borders Community Rehabilitation team caseload. Notes from the years of 2019, 2020 and 2021 of all 100 patients on the caseload were reviewed, for documentation of the following seven parameters as recommended by SIGN: ECG, blood glucose, lipid profile, prolactin, BMI/weight, smoking status and blood pressure. Results were then entered manually into a secure spreadsheet. Permission for this audit was granted by NHS Borders.

**Results:**

Initial results for the parameters of: ECG, blood glucose, lipid profile and prolactin levels demonstrate that routine monitoring of all four domains has decreased since the start of the COVID-19 pandemic. In 2019 the following numbers of patients had monitoring in these domains: ECG 56 (56%); blood glucose 84 (84%); lipid profile 74 (74%) and prolactin levels 62 (62%).

During 2020, the number of patients having monitoring in all four domains fell: ECG 31 (31%); blood glucose (72%); lipid profile 64 (64%) and prolactin levels 48 (48%). During 2021, monitoring levels remained low: ECG 30 (30%); blood glucose 71 (71%); lipid profile 62 (62%) and prolactin levels 43 (43%).

Data collection for the parameters of blood pressure, BMI/weight and smoking status is ongoing.

**Conclusion:**

Initial results indicate that the COVID-19 pandemic has negatively impacted on the routine physical health monitoring of patients under the care of the community rehabilitation team in NHS Borders. These results imply opportunities to treat and prevent conditions such as diabetes mellitus and hypercholesterolemia are being missed, further perpetuating an existing health inequality for patients with severe and enduring mental illness.

